# Mr. Chapman, on Depression of the Skull

**Published:** 1800-05

**Authors:** John Chapman

**Affiliations:** Ampthill


### *To the Editors of the Medical and Phyſical Journal.*

Gentlemen,

Since I tranſmitted you the Caſes of Injuries of the Head, wherein were related two caſes of fracture of the cranium with depreſſion,* the depreſſion ſtill remaining, I have met with another remarkable inſtance of recovery of the ſame kind, which I ſhall briefly relate, in confirmation of the opinion that the brain is capable of ſuſtaining or accommodating itſelf to a moſt extraordinary degree of preſſure, without any unpleaſant ſymptoms being produced, after the firſt ſymptoms of compreſſion have ſubſided.

W. L. a lad, ſixteen years of age, in a quarrel, received a violent blow on the right parietal bone from a hammer, which was thrown by his opponent with all his force. The claw and heel of the hammer only coming in contact with the head, the heel occaſioned a depreſſion large enough to admit a piſtol bullet, and left a bridge of undepreſſed cranium of about a quarter of an inch, from which commenced a depreſſion, occaſioned by the claw, of an inch and a half in extent, altogether producing the exact impreſſion of the ſide of the hammer. The blow knocked him down; he lay ſenſeleſs ſome minutes, and then recovered. Being alarmed at the hæmorrhage, as ſoon as he was able he went to the neareſt Apothecary to have his head dreſſed, who informed him, he thought his ſkull was fractured, and he had better go to an hoſpital. This the boy refuſed doing; his head was therefore dreſſed, very ſuperficially, four or five times, the hair not even being removed. The boy remained rather poorly four or five days, and was then left to chance, nothing more being done. Two or three months afterwards, five or ſix ſmall ſpiculæ of bone worked themſelves through the ſcalp, (the wound being healed) or were extracted by the boy's own fingers. Since this, he has not felt any farther inconvenience from the depreſſion, which remains the ſame at this moment.

In the 2d volume of Annals of Medicine, Mr. Mackie, a ſurgeon at Antigua, has related a caſe of fracture with depreſſion, together with an extenſive lacerated wound of the ſcalp, that recovered without the uſe of the trephine, and remained perfectly well to the time of his writing the account; which was upwards of thirteen months.

any

* Medical and Phyſical Journal, Numb. XI. p. 30.

The utility of publiſhing a few fortunate caſes that have eſcaped from many others that would have proved fatal, may very poſſibly be queſtioned, particularly as they do not form any decided rule of practice: — Granted. And it is to this point I wiſh to fix the attention of my profeſſional brethren. Admitting, then, that there are many caſes of fracture of the cranium with depreſſion, that will recover very well without the trephine, the natural inference is, that *many others* will occur that will do the ſame; and inſtead of uſing the trephine for fiſſures and fractures, as heretofore, it may not be wanting in half of the caſes, even when accompanied with depreſſion. The next point of inquiry then is, to aſcertain decidedly the diagnoſtic of thoſe caſes that will, and will not, recover without the uſe of the trephine. Might not this be done by a ſtrict attention to the minute ſymptoms in both caſes? If ſo, it would bring this operative part of Surgery to that certainty, wherein there could be but one opinion; and happy would it be for us, could we, in all caſes, arrive at this grand deſideratum.

I alſo take this opportunity of again adding, that I believe there are many caſes, as in Caſe 3 and 4, before related, where we may be deceived in taking an extravaſation under the ſcalp from the feel of fracture with depreſſion, for that injury, when the cranium remains ſound; and I hope to be forgiven, if I am a ſceptic to the majority of thoſe caſes called fracture with depreſſion, where the cranium is *said* to have recovered its former level. I remain,

Gentlemen,

Yours, &c.

*Ampthill,*

*April 2, 1800.*

JOHN CHAPMAN.

---

To the Editors of the Medical and Physical Journal.

Gentlemen,

IF the following Hints are conſidered worth inſerting in any part of your Journal, they are much at your ſervice.

John Chapman.

A Lady, who, during ſeveral different periods of pregnancy, was much teaſed with a ptyaliſm, lately upon her recovery from typhus, had a return of this troubleſome affection, for which the uſe of diuretics was ſuggeſted to her. She eat very liberally of celery, which had a diuretic effect, and entirely removed the ptyaliſm. How far might diuretics, then, be of ſervice in removing the ptyaliſm brought on by mercury? and, vice verſa?

verfa? As thefe fecretions appear to fympathize (if I may be allowed the expreffion) with each other, might not a falivation, continued for fome time, cure diabetes? From the vifcidity of the faliva in thefe cafes, I fhould conceive, a priori, it would be of fervice.

I am refpectably informed, that the Chinefe are very fuccefsful in removing difeafes by attending to the fympathy of parts; and if it was more attended to in this country, probably fome good might be derived.

